# Object Detection and Information Perception by Fusing YOLO-SCG and Point Cloud Clustering

**DOI:** 10.3390/s24165357

**Published:** 2024-08-19

**Authors:** Chunyang Liu, Zhixin Zhao, Yifei Zhou, Lin Ma, Xin Sui, Yan Huang, Xiaokang Yang, Xiqiang Ma

**Affiliations:** 1 School of Mechatronics Engineering, Henan University of Science and Technology, Luoyang 471003, China; 2 Longmen Laboratory, Luoyang 471003, China; 3 Key Laboratory of Mechanical Design and Transmission System of Henan Province, Luoyang 471003, China

**Keywords:** fusion perception, object detection, YOLO-SCG, point cloud clustering

## Abstract

Robots need to sense information about the external environment before moving, which helps them to recognize and understand their surroundings so that they can plan safe and effective paths and avoid obstacles. Conventional algorithms using a single sensor cannot obtain enough information and lack real-time capabilities. To solve these problems, we propose an information perception algorithm with vision as the core and the fusion of LiDAR. Regarding vision, we propose the YOLO-SCG model, which is able to detect objects faster and more accurately. When processing point clouds, we integrate the detection results of vision for local clustering, improving both the processing speed of the point cloud and the detection effectiveness. Experiments verify that our proposed YOLO-SCG algorithm improves accuracy by 4.06% and detection speed by 7.81% compared to YOLOv9, and our algorithm excels in distinguishing different objects in the clustering of point clouds.

## 1. Introduction

Information perception is a key technology that supports mobile robots to realize autonomous, safe and efficient actions in complex environments, mainly including perception methods such as vision, radar and multi-sensor fusion.

Vision sensors are able to acquire information such as the category, color and texture of external objects, with the category being particularly important. Object detection often employs the YOLO (You Only Look Once) [1] algorithm, which is the first end-to-end network framework enabling real-time detection. It uses regression to optimize solutions for category and location as a whole. The method enables real-time monitoring, but its oversimplified structure brings lower accuracy. In order to solve this problem, the YOLO series is constantly updated and iterated, deriving a series of improved models [2,3,4]. For instance, YOLOv9 [4] relies on Generalized Efficient Layer Aggregation Network (GELAN) and Programmable Gradient Information (PGI) to obtain higher accuracy and speed among similar models. However, focusing solely on global information or local features in the object detection process can easily lead to problems such as reduced recognition rate and missed detection. For example, the multimodal method [5] obtains final results by training fusion units to acquire representation vectors that fuse image features and semantic features, while focusing too much on global information can make it difficult to infer the correct answers. On the other hand, overemphasizing local information limits the model’s ability to acquire global information. Object detection algorithms such as YOLOv9 divide images into a number of grids to extract features separately, which can easily result in the missed detection of distant small objects or overlapping objects. Therefore, introducing an attention mechanism can enhance the ability to focus on local features while simultaneously capturing global features and local information. This approach allows for flexible allocation of attention weights to enhance model performance and improve information capture abilities [6].

The attention mechanism is a technique that mimics human ability to selectively observe and focus on key information according to need while ignoring the rest [5]. It aggregates algorithms to the most relevant parts of the input based on their relative importance, tending to focus on unique aspects when processing large amounts of information [7]. Currently, the attention mechanism is divided into the channel attention mechanism [8], hybrid attention mechanism [9], self-attention mechanism [10], etc. SENetV2 establishes an attention mechanism based on channel correlation, which automatically obtains the importance of the feature channels and rationally assigns different weights according to the importance of each channel feature to reduce feature redundancy [11]. Chen [8] introduced Efficient Multi-Scale Attention (EMA), a multi-scaled channel attention mechanism, into YOLOv7, which aggregates pixel-level features and reduces missed detections and false detection through cross-channel interaction. In order to enhance the accuracy of pipeline leakage detection, Peng et al. [9] integrated Convolutional Block Attention Module (CBAM) into YOLOv5, which enables the model to focus on extracting features, attenuating the influence of complex backgrounds and reducing computational amount by combining with adaptive spatial feature fusion. Cao et al. [10] introduced the adaptive attention mechanism Swin Transform (SWT) into YOLOv7 to reduce the computation load. Swin Transformer is an adaptive attention mechanism that integrates into a convolutional neural network to guide the network to focus on contextual spatial information and improve its sensitivity to small objects. Wu et al. [12] extended the perceptual domain of the model in YOLOX using attention modules, resulting in an average accuracy improvement of approximately 4.24% compared with the pre-optimization period. Therefore, this paper introduces SENetV2 into the convolutional neural network to improve its leakage detection of small objects or overlapping objects. At the same time, Context Guided Block, a lightweight semantic network, replaces the convolutional module to solve the problems of parameter numbers and the increase in training cost caused by the multi-branch structure of SENetV2 while ensuring the recognition accuracy.

After completing the target detection for vision, the processing of the detected target can obtain the color, texture and other information of the object in order to achieve finer information perception. However, the above methods cannot directly provide depth information, are greatly affected by occlusion and cannot directly perceive the shape and size of the object, so there is a big limitation in using only a single 2D camera as an information perception element. Li et al. [13] combined 2D detection boxes from multiple cameras to approximate 3D detection box for the depth information. Ding et al. [14] designed a motion target localization algorithm by improving the optical flow method, and then reconstructed the 3D coordinates of points within the motion target area based on the parallel structure of binocular vision. LiDAR can provide accurate distance information and accurate perception of object shape. The processing of radar point clouds is a commonly used clustering method [15,16] which is flexible but sensitive to model parameters, data noise and outliers. The use of a convolutional neural network point cloud processing algorithm can improve the detection accuracy and robustness [17,18], but it is prone to problems such as large computational volume and poor real-time algorithm performance. In order to improve the training and inference speed, some studies have voxelized the point cloud [19] or used sparse convolution [20], but both methods cause permanent loss of information and are only applicable for special scenarios. Although radar-based environment sensing can effectively determine the spatial location of an object, the large amount of point cloud data and the inability to obtain the color and texture information of the object make it difficult to use for information sensing alone.

In summary, existing single-sensor methods have significant limitations in environmental perception. While visual cameras can provide color and texture information of objects and LIDAR provides accurate distance and shape perception, methods relying on one type of sensor cannot comprehensively understand and percept targets in complex scenes. Therefore, in order to address the limitations of single sensors such as camera and radar, the use of fusion can effectively improve the information perception. Veitch-Michaelis J. [21] data-fused LiDAR point cloud data with parallax maps obtained by stereo matching which realized simple calibration of camera and radar, but in low texture regions, the information perception ability will be reduced dramatically. Chen [22] proposed MV3D (Multi-View 3D Object Detection Network) to fuse LiDAR point cloud data with RGB image information for predicting directional 3D bounding boxes. Although MV3D streamlines the VGG16 network, the algorithm recognition time is as high as 0.36 s per frame, which makes it difficult to achieve real-time processing. Some works have optimized MV3D by introducing an encoder-decoder backbone [23] or a voxel feature encoding (VFE) layer [24], which has advantages in both accuracy and detection speed. In addition, Duan [25], Liu [26] and others have recently used attention mechanisms to fuse data from LiDAR and cameras, achieving competitive 3D object detection performance.

All of the above works are oriented towards 3D object detection. The fusion of LiDAR and the camera is also capable of achieving accurate depth measurement, information perception and other tasks. Battrawy R. [27] proposed a method to fuse LiDAR with images obtained from stereo matching for dense scene traffic estimation. Wang [28] utilized Convolutional Neural Networks (CNNs) for the fusion of radar and camera data to achieve object detection based on depth measurements; although the algorithm had a significant improvement in detection speed, a sparse non-zippered pooling layer was constructed before converting to bird’s-eye view, which lost a large amount of information. Varuna [29] utilized the fusion of camera and radar information for the environment sensing of unmanned vehicles, which was combined with the application of a wide-angle camera to an autonomous mobile robot and the establishment of a sensor fusion framework, which improves the unmanned vehicle’s ability to perceive environmental information, but the proposed method is only applicable to a single image and cannot be used for data streaming. Wang H. [30] used LiDAR point clouds to obtain regions of interest by clustering, projected the regions of interest onto the image and used a YOLOv3 object detection algorithm to detect the object on the image corresponding to the candidate region, and the proposed algorithm’s processing time of each image frame reached 0.09 s, but due to the limitation of the underlying model, it only has 69.36% accuracy (Easy) on the KITTI dataset. Wang [31] proposed Bi-Consistency Guidance Incomplete Multi-view Clustering when using clustering methods to deal with incomplete data. The method focuses on identifying instances and clustering optimization from multi-view data. While these studies confirmed the feasibility of combining LiDAR and cameras, several challenges emerge when applying the algorithms for a broader range, such as the complexity of processing high-dimensional point cloud data and the algorithms’ limited accuracy. A promising direction for future research is upgrading deep neural networks with new architectures and attention mechanisms, which may achieve more accurate and efficient perception.

Based on the above analysis, this paper proposes an information sensing system based on multi-sensor fusion. Camera and LiDAR are used to acquire color images and depth images of the outside world, respectively, and with vision as the core, the information perception is accomplished by improving the accuracy of the object detection algorithm, reducing the data processing needs of the LiDAR point cloud and fusing the data acquired by the two sensors at the data level. The contributions of this paper are as follows:

1. We propose an object detection model, YOLO-SCG, which integrates the latest SENetV2 attention mechanism, which can effectively improve the detection ability of the model, in addition to replacing the convolution in YOLOv9 with the Context Guided Block that can simulate the human visual system relying on contextual information, which can effectively improve the speed of the model.
2. It is experimentally verified that the YOLO-SCG proposed in this paper has higher accuracy and excellent real-time performance compared with the current popular target detection models.
3. We propose an algorithm with visual detection as the core and radar assistance for joint information perception. We try to incorporate the image detection information into the point cloud to obtain finer 3D information. Finally, the effectiveness of the proposed fusion method for information perception is verified by experiments.

## 2. Integration Programs and Models

**Figure 1:**
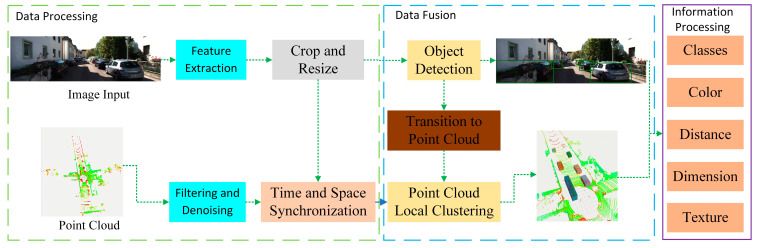
Multi-sensor information fusion model.

Figure 1 shows our method for integrating visual and point cloud data. We have performed basic processing on the input images and point cloud data, including feature extraction and cropping for images, filtering of point clouds and removal of ground points, which can simplify data processing and enhance algorithm stability. At the end of data processing, we synchronized the space and time of the image and point cloud data. In the second part, we first use YOLO-SCG for object detection on the input feature map, obtaining essential object coordinate frame information, which transform it into the point cloud coordinate system. Then, we perform Local Euclidean clustering on the point cloud according to the information detected in the image. At the end of this part, we obtain both two-dimensional and three-dimensional information about external objects. Finally, after simple processing of this information, we can obtain category, color, clustering, texture, size and so on.

### 2.1. YOLO-SCG Object Detection Model

**Figure 2:**
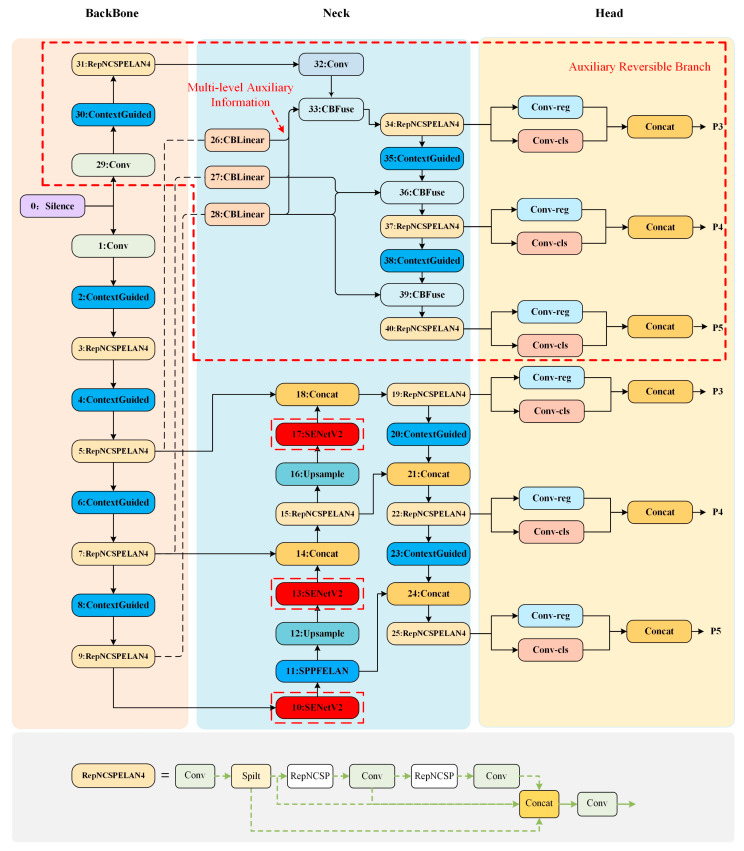
YOLO-SCG network structure diagram.

The network structure of Programmable Gradient Information (PGI) and Generalized Efficient Layer Aggregation Network (Generalized ELAN), proposed by YOLOv9, is shown in Figure 2. This aims to reduce the problem of information loss due to network deepening by generating reliable gradient information and updating the network parameters.

However, YOLOv9 pays more attention to local information when performing feature extraction, which limits the model’s ability to obtain global information, so it can better fuse local and global information by replacing the convolutional layer in the original network to strengthen the network’s ability of feature extraction and improve the model’s detection performance. However, when recognizing overlapping or distant objects, its detection accuracy reduces slightly. The introduction of the attention mechanism can filter out irrelevant external information so that the model can focus on processing the key information of the input data, and improve the model classification accuracy and recognition detection accuracy. Therefore, based on the architecture of YOLOv9, we propose a new object detection model, YOLO-SCG, to improve its classification accuracy and object detection accuracy. The proposed model incorporates both the SENetV2 attention mechanism and the Context Guided module, which will be described in detail in the following subsections.

The network structure diagram of YOLO-SCG is shown in Figure 2. The Context Guided module is used to replace the Conv layer in the original network, and the purpose of improving the accuracy of its semantic segmentation is realized by fusing the information. After preprocessing, the image is sent to the backbone part of the network to extract different feature information and semantic information, and then the extracted features are passed to the neck (Neck) for feature fusion; the main structure of the neck remains unchanged, and the SENetV2 attention mechanism is added to the neck in order to improve the classification accuracy of the model.

#### 2.1.1. SENetV2 Module

**Figure 3:**
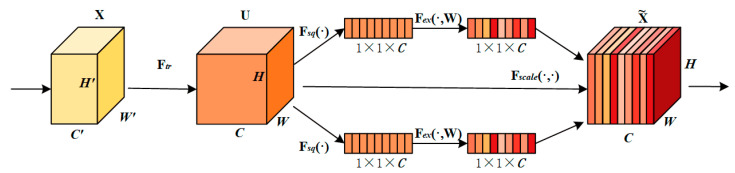
SENetV2 structure diagram.

SENetV2 is an attention mechanism that improves classification accuracy by adaptively adjusting the channel relationships of a convolutional network. The model uses a multi-branch fully connected layer for squeezing and excitation operations to enable the network to learn different features of the input data more efficiently, taking into account the interdependence between channels and global information, and its structure is shown in Figure 3.

The feature map U is generated after the convolution operation F*_tr_*, which is described by Equation (1). V is introduced to denote the filter kernel, $v_{c}$ is the parameter of the Cth filter, $v_{C}^{S}$ denotes its single channel acting on the corresponding channel X, * represents the convolution operation and the output is generated by summing over all channels.

(1) $$u_{c}=v_{c}*X=\sum_{S=1}^{C^{\prime }}v_{C}^{S}*X^{S}$$

**Figure 4:**
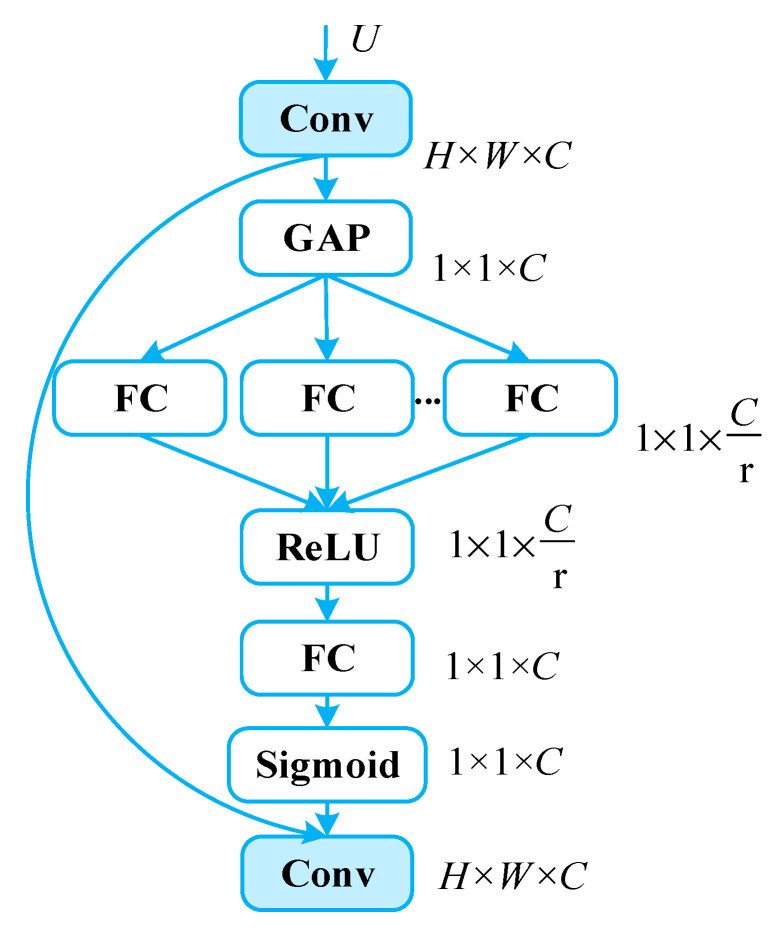
SaE module.

The attention mechanism employed by this work mainly uses the Squeeze aggregated Excitation (SaE) module, which combines squeeze excitation operations and a multi-branch fully connected layer to dynamically adjust channel importance by adjusting channel weights. Figure 4 displays the internal mechanism.

Equation (2) illustrates the global average pooling operation to compress the feature map U into 1 × 1 × C channel descriptors. By converting each channel’s spatial position information into a single scalar value, the operation shields the spatial distribution information and stresses the correlations of inter channels.

(2) $$z_{c}=F_{sq}\left(u_{c}\right)=\frac{1}{H\times W}\sum_{i=1}^{H}\sum_{j=1}^{W}u_{c}(i,j)$$

The output obtained through global average pooling is fed into the multi-branch fully connected (FC) layer. The process serves two purposes: firstly, it learns the correlations between channels to assign corresponding weights, enhancing the model’s perception of different channel information. Secondly, it reduces the channel dimensionality to decrease computational load, while employing ReLU functions to constrain model complexity. The second fully connected layer can be used to restore the original channel dimension. Employing the Sigmoid activation function helps in learning nonlinear interactions between channels and non-exclusive relationships among multiple channels, effectively capturing channel dependencies. Meanwhile, the output represents the weights of each channel’s importance, as illustrated in Equation (3). Finally, according to Equation (4), the obtained C weights are multiplied with the C channels of feature map U, reassigning the channels’ importance in feature maps accordingly.

(3) $$s=F_{ex}\left(z,W\right)=\sigma \left(g\left(z,W\right)\right)=\sigma \left(W_{2}\delta \left(\sum W_{1}z\right)\right)$$

(4) $$\tilde{x_{c}}=F_{scale}\left(u_{c},s_{c}\right)=s_{c}\cdot u_{c}$$

#### 2.1.2. Context Guided Model

**Figure 5:**
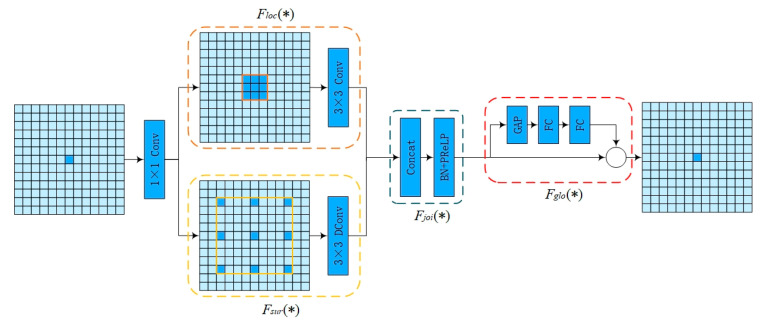
Context Guided Block (CG block).

Context Guided Block (CG block) is a lightweight and efficient semantic segmentation network, which mainly consists of a local feature extractor $F_{loc}$(*), a surrounding context extractor $F_{sur}$(*), a joint feature extractor $F_{joi}$(*) and a global context extractor $F_{glo}$(*), as shown in Figure 5.

Equation (5) shows the computational process of joint local and contextual information. Specifically, the input feature map is first halved in the number of channels by 1 × 1 convolution. This output is then fed to the local feature extractor for channel-level convolution operations to obtain local feature information; at the same time, this output is also fed to the surrounding context extractor to increase the size of the receptive field using null convolution to extract broader contextual information. Next, the joint feature extractor performs channel splicing of the local and contextual information to ensure that the model understands the information in each pixel or localized region and the relationship of these regions in the whole.

(5) $$X_{c}=F_{joi}(F_{sur}\left({Conv}_{1\times 1}\left(X\right)\right)+F_{loc}\left({Conv}_{1\times 1}\left(X\right)\right))$$

Equation (6) illustrates the computational process of extracting the global information of the whole image. The obtained joint feature information is taken as an input, and the feature information is dimensionally reduced after the global average pooling operation. After that, the feature information is further processed through two fully connected layers. Finally, the processed features multiply with the original input to enhance and refine the global contextual feature information, which helps the network to better understand the global information of the whole image.

(6) $$F_{glo}=X_{c}\times (F_{c}\left(GAP\left(X_{c}\right)\right))$$

### 2.2. Time and Space Are Synchronized

**Figure 6:**
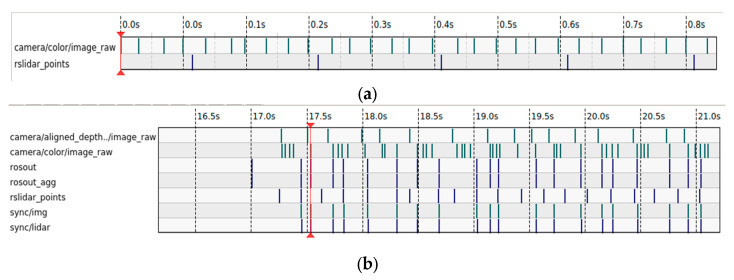
Data information before and after time synchronization. (**a**) Raw data information. (**b**) Post-synchronization data information.

In this paper, the camera used has an output frame rate of 30 FPS, while the LiDAR has an output frequency of 20 Hz. This means the camera’s sampling period is 33.3 ms and the LiDAR’s sampling period is 50 ms. When using the ROS system for data fusion between the LiDAR and the camera, different types of data from the camera and LiDAR are received. The corresponding information is only output when messages are received from each source with the same timestamp, thus achieving message synchronization output and sensor data time synchronization. Figure 6a,b. show the data frequency before and after time synchronization.

The coordinate system transformation between LiDAR and camera falls under rigid body transformation, where the objects in the coordinate system are not deformed by the transformation but only need to be rotated and translated. The KITTI dataset provides the relevant transformation matrices, so it is only necessary to use the matrices to convert the point cloud and image coordinate systems by translation and rotation.

When performing the transformation of the coordinate system, the rotation matrix formula is as follows:

(7) $$R=R_{z}*R_{y}*R_{x}\;=\left[\begin{array}{ccc} cos\alpha cos\beta & cos\alpha sin\beta sin\gamma -sin\alpha cos\gamma & cos\alpha sin\beta cos\gamma +sin\alpha sin\gamma \\ sin\alpha sin\beta & sin\alpha sin\beta sin\gamma +cos\alpha cos\gamma & sin\alpha sin\beta cos\gamma -cos\alpha sin\gamma \\ -sin\beta & cos\beta sin\gamma & cos\beta cos\gamma \end{array}\right]$$

Expressing the point $P_{l}(x_{l},y_{l},z_{l})$ in the LiDAR coordinate system in chi-square form is transformed to the point $P_{l}(x_{c},y_{c},z_{c})$ in the camera coordinate system as follows:

(8) $$\left[\begin{array}{c} x_{c}\\ y_{c}\\ z_{c}\\ 1 \end{array}\right]=\left[\begin{array}{cc} R & T\\ 0 & 1 \end{array}\right]\left[\begin{array}{c} x_{l}\\ y_{l}\\ z_{l}\\ 1 \end{array}\right]$$

where T is the translation matrix and $\left[\begin{array}{cc} R & T\\ 0 & 1 \end{array}\right]$ is the transformation matrix between the LiDAR coordinate system and the camera coordinate system, which is provided by the KITTI data, and is used in this paper for the transformation between coordinate systems.

### 2.3. Euclidean Clustering and Bracket Box Construction

Euclidean clustering is an algorithm implemented based on the Euclidean distance measurement. It uses a K-dimensional tree (Kd-tree) to calculate the Euclidean distance between the point cloud. Based on the calculation results, it finds the closest neighboring points, and the point cloud that is within a fixed threshold represents a subset. The role of Kd-tree is to improve the search speed. The Euclidean distance between points $\left(x_{1},y_{1},z_{1}\right)$ and $\left(x_{2},y_{2},z_{2}\right)$ in space is calculated as Equation (9):

(9) $$d=\sqrt{(x_{1}-x_{2}{)}^{2}+(y_{1}-y_{2}{)}^{2}+(z_{1}-z_{2}{)}^{2}}$$

After the point cloud clustering, a bounding box model is used to describe the shape of the obstacle point cloud. We use the AABB (Axis-Aligned Bounding Box) algorithm for this purpose. In the AABB model, each edge is parallel to a coordinate plane. The dimensions of the rectangular bounding box in 3D dimensions can be different and may not necessarily be cubic. The AABB bounding box contains two important vertices, $P_{min}=\left[x_{min},y_{min},z_{min}\right]$ and $P_{max}=\left[x_{max},y_{max},z_{max}\right]$, whose three-dimensional dimensions are respectively computed as in the following Equations:

(10) $$L=x_{max}-x_{min}$$

(11) $$W=y_{max}-y_{min}$$

(12) $$H=z_{max}-z_{min}$$

where L, W and H are length, width and height, respectively; if W > L, then exchange the corresponding values to get the size of the obstacle-enclosing box. In the real-world environment, the coordinate axes may not always be parallel to the surrounding obstacles; in this case, AABB will surround all the point clouds of this obstacle.

## 3. Experimentation and Analysis

### 3.1. Hardware Configuration and Evaluation Metrics

The system used in this experiment is Ubuntu 18.04, the GPU is GeForce RTX2080Ti, memory is 32 G, CUDA 10.2 and Python 3.9. During the training procedure, the batch size is 4, while the learning rate is 0.01.

The performance evaluation metrics used in this paper for the object detection task are precision Equation (13), recall Equation (14) and mean average precision Equation (15). In addition, we added the size of the parameters of the model and the amount of computation to the evaluation metrics. It was computed for the convolutional layer, the fully connected layer and the other layers in the network structure that have parameters.

(13) $$Precision=\frac{TP}{TP+FP}$$

(14) $$Recall=\frac{TP}{TP+FN}$$

(15) $$AP=\int_{0}^{1}Precision(Recall)d(Recall)$$

(16) $$mAP=\frac{\sum_{i=0}^{i=n}AP}{n}$$

where TP is the number of positive samples predicted to be positive, FP is the number of negative samples predicted to be positive, FN is the number of negative samples predicted to be negative and AP is the average accuracy.

### 3.2. Datasets

For this experiment, the VOC 2007 datasets was chosen for training to detect as many classes as possible in an open environment. VOC, the full name of which is Visual Object Classes, is a dataset provided by the PASCAL VOC project for object detection and image classification. It consists of 9963 images, with horizontal resolutions ranging approximately 375~500 and vertical resolutions around 350~500. The dataset covers 20 different types of objects, including common targets such as “aircraft”, “bicycle”, “person”, etc. The scene in the dataset is complex, with large differences between similar targets in a single image and varying degrees of occlusion. Therefore, using this dataset for training can improve the model’s generalization ability. The current study employs a split ratio of 6:3:1 to divide the training set, testing set and validation set.

For the radar-related experiments, since the VOC dataset only contains image data, it is necessary to use the KITTI dataset for additional testing. The KITTI dataset is widely used for research in autonomous driving and computer vision, created by the Karlsruhe Institute of Technology and the University of Stuttgart. The dataset provides rich sensor data, including high-resolution images, LiDAR point clouds, GPS/IMU data and vehicle attitude information, covering various urban environments such as city streets and rural roads.

### 3.3. YOLO-SCG Ablation Experiment

To investigate the resulting enhancement effects of integrating the SENetV2 attention mechanism and the Context Guided block into the YOLOv9 network and to evaluate whether these two methods outperform other attention mechanisms and convolutional blocks, we designed ablation experiments. We conducted comparative analyses of the effects produced by incorporating various attention mechanisms and convolutional blocks into the network structure. We made various modifications to YOLOv9 and tested them using different configurations, with each experimental setup corresponding to a comprehensive set of evaluation metrics.

#### 3.3.1. Comparison of Different Attention Mechanisms

**Figure 7:**
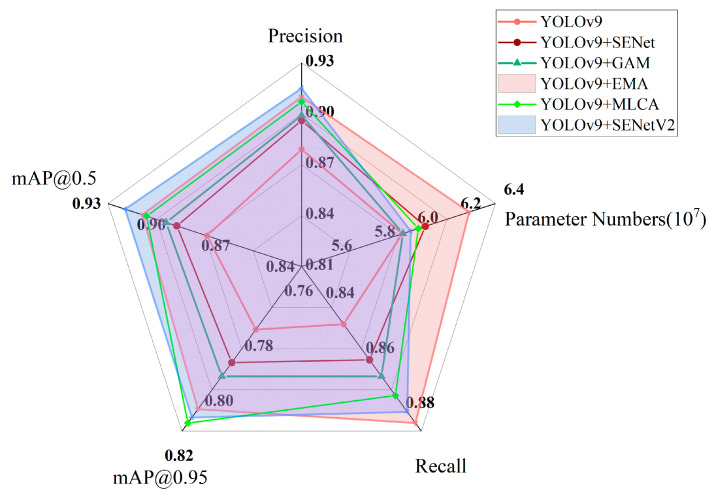
Parameter map of ablation experiment-attention mechanism.

Figure 7 shows the results of the ablation experiment after integrating various attention mechanisms into YOLOv9.

Integrating SENet (Squeeze and Excitation Networks) and SENetV2 into YOLOv9 can increase object detection accuracy via introducing squeeze excitation operations. The feature maps can be processed by dynamically adjusting the importance of different channels. Compared with SENet, SENetV2 further integrates a multi-branch fully connected layer to learn the correlation between these channels, resulting in a 4.29% increase in mAP(mean Average Precision), but at the same time leads to a 0.6% increase in parameter numbers.

We also investigated introducing the local average pooling and global average pooling operations of Mixed Local Channel Attention (MLCA) into YOLOv9. Firstly, it extracts local spatial information through local pooling. Subsequently, it introduces two branches: the first branch obtains global information by global average pooling, while the second branch uses convolution operations to capture local interaction information between channels and leverage useful features. Furthermore, fusing two branches obtains global contextual information, resulting in a 3.16% increase in detection accuracy but raising the computation complexity, with an increase of 1.23% in parameters.

The introduction of EMA in YOLOv9 improves target detection accuracy by both reshaping some channels of the feature map into batch dimension and dividing the channel dimension into multiple sub-features to ensure uniform distribution of channel information, as well as encoding the global information in parallel and capturing pixel-level relationships across the latitudinal interaction module, resulting in 3.39% increase in detection accuracy. However, due to its parallel branches, its parameters increased by 5.12%.

Global Attention Mechanism (GAM) is integrated into YOLOv9, and its 3D alignment is introduced to preserve global feature information across three dimensions, while the channel dependency across latitude is enhanced through Multilayer Perceptron (MLP); this maintains better global consistency, but it pays less attention to local features, resulting in only a 0.05% increase in detection accuracy.

A comprehensive analysis shows that the incorporation of the SENetV2 attention mechanism produces a better enhancement that not only improves the model performance but also achieves the overall best performance in terms of all evaluation metrics.

#### 3.3.2. Comparison of Different Module Replacement Convolutions

The experimental results of replacing the convolutional layer using different modules are shown in Table 1. The Context Guided self-attention mechanism is used to replace the convolutional layer of the original network, which fuses local features, surrounding context and global context information to enhance the capturing of the relationship between the object and the background and improve the accuracy of object detection. Experimental results show that this method significantly improves the accuracy of object detection, with a 4.18% improvement in mAP, while the amount of parameters is reduced by 8.06%. We also use three convolution modules, DynamicConv, DualConv, and SPD-Conv, respectively, to replace the convolution layers. DynamicConv improves the model performance by dynamically adjusting the convolution kernel but also increases the model parameter count and computational complexity. DualConv uses two convolutional kernels for convolution operation to optimize the information processing and feature extraction capability, which reduces the amount of model parameters by 8.8%, but the detection accuracy is not as good as Context Guided due to the redundancy of information. SPD-Conv (Space-to-Depth Convolution) captures multi-scale features and increases the receptive field by using different dilution rates. However, its complex structure leads to longer training times and a higher risk of overfitting. Comparative analysis reveals that replacing the convolutional layer using the Context Guided attention mechanism meets the requirements in all aspects of performance.

### 3.4. YOLO-SCG Comparison Experiment

**Figure 8:**
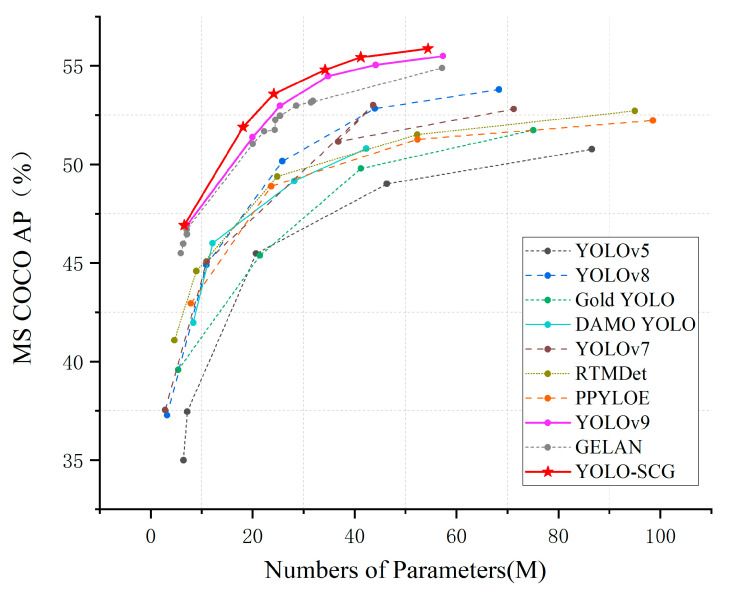
Comparison of real-time object detectors on MS COCO dataset.

In this section, the comparison experiments of the proposed model with other models are given, and the specific data is shown in Table 2. The proposed model in this paper has better performance in terms of precision, recall, and F1_score. Due to the incorporation of the Context Guided lightweight network, the model reduces the number of parameters. For mAP@0.5, YOLO-SCG (0.880) reduces the value by 0.45% with respect to the original YOLOv9 (0.884) model, and improves the value by 6.54% with respect to the more popular YOLOv5s (0.826). However, in a comparison of mAP@0.95, the value improved by 2.3% compared to YOLOv10s, and YOLO-SCG improved the value by 4.06% relative to the original YOLOv9. This indicates that while the model’s performance is comparable to the original model in detecting regular objects, YOLO-SCG maintains high detection accuracy in situations involving small targets and overlapping objects. In addition, we compared our model with other real-time object detectors on the MS COCO dataset. As shown in Figure 8, our model exhibits higher accuracy than other models with the same number of parameters.

### 3.5. Information Acquisition by Multi-Sensor Fusion

Cameras are greatly affected by light, which may lead to image quality degradation under different lighting conditions and make it difficult to directly obtain the distance information of objects, posing challenges for depth perception and 3D reconstruction. In this paper, we try to perform object detection on the image and then perform local clustering on the point cloud according to the obtained results, aiming to get a more accurate and fast information perception model.

#### 3.5.1. Robustness Evaluation of Object Detection Model

**Figure 9:**
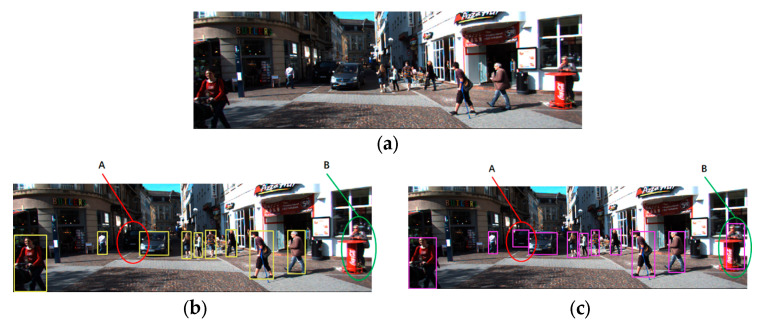
Object detection effect comparison. (**a**) Original map. (**b**) YOLOv9 detection. (**c**) YOLO-SCG detection.

The detection effect of the proposed YOLO-SCG object detection algorithm is shown in Figure 9. As seen in areas A and B of the figure, although the YOLOv9 object detection algorithm achieves better accuracy, it still misses detection in some extreme cases (such as occlusion or low light). However, YOLO-SCG is sufficient to eliminate this leakage phenomenon after ensuring the detection effect of the original model.

**Figure 10:**
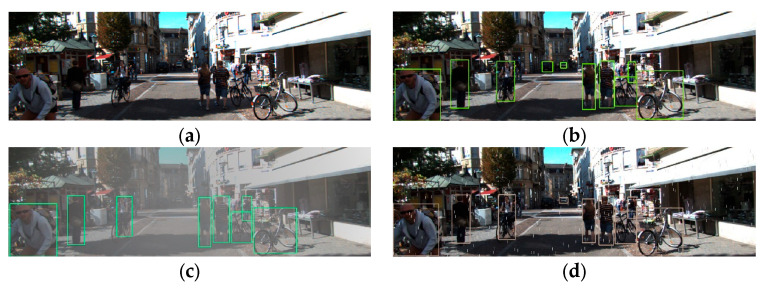
Robustness testing. (**a**) Original image. (**b**) Normal environment detection. (**c**) Simulated fog detection. (**d**) Simulated rain detection.

To evaluate the robustness of the model under external environmental disturbances, we conducted experiments simulating various conditions. For images of the same scene, 10, 8 and 9 are detected in a normal environment, simulated foggy day and rainy day, respectively. Among them, Figure 10b shows the detection effect in a normal environment, which shows that the model can clearly recognize both distant and near objects. Figure 10c,d simulates detection in foggy and rainy conditions, and it can be seen that the proposed model is able to recognize all the objects at normal distances regardless of the weather. However, detecting distant objects poses some challenges in heavy rain and thick fog scenarios, resulting in 1–2 instances of missed detections. Nevertheless, this challenge remains within acceptable limits.

#### 3.5.2. Evaluation of the Effectiveness of Localized Euclidean Clustering

**Figure 11:**
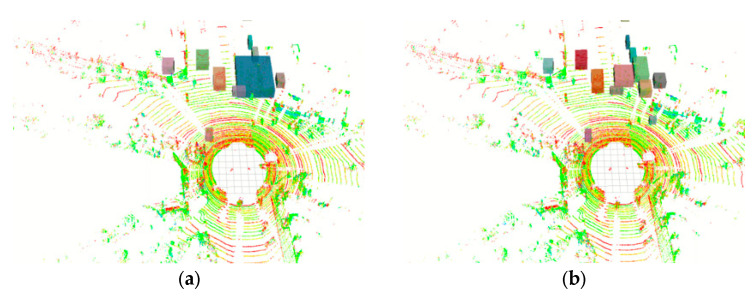
Comparison of point cloud clustering effect. (**a**) Euclidean clustering method. (**b**) Fusion clustering method.

Next, this paper uses conventional Euclidean clustering as a comparison, as shown in Figure 11a. When dealing with independent objects (those that are far away from other objects), clustering methods can successfully distinguish between different objects. However, when the objects are close to each other or overlap, clustering the point cloud data is unable to distinguish different objects. More seriously, the algorithm will categorize different objects as a single object. This may result in failing to recognize movable objects or recognizing stationary objects as moving. The clustering effect using our proposed method is shown in Figure 11b, where vision-based object detection obtains the detection frame and transforms it to the point cloud coordinate system, followed by performing Euclidean clustering within the coordinate frame. This method effectively enhances the refinement of clustering and reduce the number of point clouds to be clustered.

**Figure 12:**
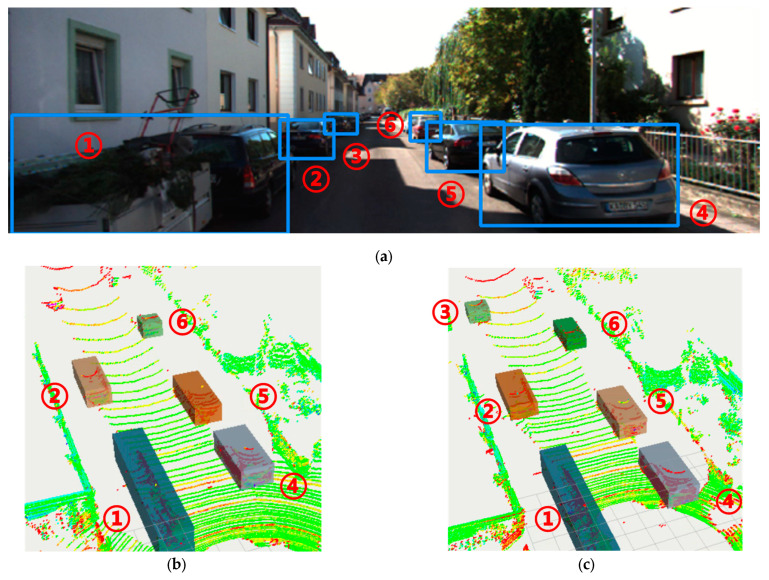
Correspondence between object detection and clustering. (**a**) YOLO-SCG detection effect. (**b**) Region Growth clustering separately. (**c**) The proposed method integrated clustering.

Figure 12 compares the results of the proposed method and two clustering algorithms for point clouds. Figure 12a shows the detection effect of YOLO-SCG, which recognizes six cars in the image despite the large occlusion produced; Figure 12b shows the effect of clustering using Region Growing alone, in which the method fails to cluster the object at distant locations. Figure 12c depicts that integrating YOLO-SCG with Euclidean Clustering can recognize six cars more accurately.

#### 3.5.3. Multi-Sensor Fusion for Information Perception

The above experiments evaluated and verified the advantages of our proposed object detection algorithm and the feasibility of the multi-sensor fusion algorithm. Subsequently, there is a need to organize and summarize the collected information. In Figure 11, the visual detection of objects in front mainly detects cars and pedestrians; after local point cloud clustering of the point cloud within the same field of view as the camera, we are able to obtain distance and size information about the objects. The information perceived for pedestrians in the A and B areas in Figure 11 is shown in Table 3, which mainly includes the information of category, distance, size, color and texture.

To test the effectiveness of the proposed multi-sensor fusion algorithm, we conducted a comparative study using 30 consecutive frames of fused data, focusing on distant cars as an example. We statistically compared the performance of our improved information perception algorithm against the traditional Euclidean clustering algorithm in measuring errors of typical values of the actual size of objects in the environment. In order to evaluate the proposed method, we compared it with three methods, namely Euclidean clustering, Region Growing and Hierarchical Clustering. Table 4 shows the four methods’ average relative measurement errors and leakage rates. Since visual object detection is introduced as an a priori frame followed by clustering, the proposed method in this paper has a smaller error in all the metrics compared to the traditional clustering method. In Table 4, the Euclidean clustering method resulted in a miss detection rate of 23.3%, and the Region Growing and Hierarchical Clustering generated 13.3% and 16.6% missed frames, respectively, which include undetected objects and merged objects. In contrast, our method failed for two frames. Regarding perception speed, Hierarchical Clustering’s detection speed is 22 fps since it requires a complete hierarchy structure and involves similarity calculation. Using YOLO-SCG as prior information for Euclidean Clustering is 32.2% faster than normal Euclidean Clustering. In general, this method improves the efficiency, accuracy and stability of clustering.

In 30 frames of detection, the Euclidean clustering method resulted in a miss detection rate of 23.3%, which include undetected objects, merging with other objects, etc.; in contrast, our method failed to recognize only 2 frames.

## 4. Conclusions

In this paper, we propose an information perception model that integrates image-based object detection with point cloud clustering to achieve enhanced multi-information perception of surrounding objects. For the object detection aspect of images, YOLO-SCG, an improved object detection model based on YOLOv9, is proposed. This model combines the latest attention mechanism and lightweight convolution, maintaining higher detection accuracy while reducing the number of parameters (11.8%). The final detection accuracy of the model can reach 0.846 mAP@0.95, which is higher than that of YOLOv7, YOLOv9 and other models. We also use object detection data to aid in local clustering of point clouds. YOLO-SCG is used to detect objects in front of us, and the detection results are transmitted to the point cloud for local clustering, finally obtaining more accurate clustering effects.

In the future, our work will focus on optimizing the fusion algorithms for LiDAR and cameras. To combine the advantages of the two sensors, we need to explore and optimize fusion methods for better information perception. However, a challenge emerges in fusing these heterogeneous data with consistency and reliability due to the different radar and camera data formats. Overcoming this challenge could enhance the radar-camera data fusion and obtain more accurate and comprehensive information in complex environments.

## Figures and Tables

**Table 1:** Ablation experiments-comparison of different convolutions.

Model	P	R	mAP		Parameter Numbers
			0.5	0.5:0.95	
YOLOv9	0.879	0.851	0.884	0.783	60,678,400
YOLOv9 + DynamicConv	0.899	0.869	0.904	0.806	60,925,694
YOLOv9 + DualConv	**0.914**	0.878	0.913	0.808	**55,290,560**
YOLOv9 + SPD-Conv	0.907	**0.887**	0.915	0.810	56,740,232
YOLOv9 + Context Guided	0.911	0.885	**0.921**	**0.815**	55,456,256

**Table 2:** Comparison of different models.

Model	P	R	F1_Score	mAP		Parameter Numbers	FPS
				0.5	0.5:0.95		
Faster-RCNN	0.742	0.680	0.716	0.763	0.427	——	19
YOLOv5s	0.782	0.789	0.724	0.826	0.640	**7,089,004**	55
YOLOv7	0.839	0.829	0.831	0.850	0.669	36,512,236	71
YOLOv9	0.879	0.851	0.868	0.884	0.813	60,678,400	64
YOLOv10s	0.903	0.881	0.889	**0.885**	0.826	8,096,880	66
YOLO-SCG	**0.914**	**0.886**	**0.892**	0.880	**0.846**	57,515,194	69

**Table 3:** Access to information display.

Class	Distance (m)	Sizes (m)	Color
Car	9.43	2.17 ×4.77 × 1.61	Black
Person	5.51	0.72 × 0.33 × 1.5	Red

**Table 4:** Average relative error and leakage rate.

Methodologies	Distance (m)	Length (m)	Width (m)	Height (m)	Leakage Rate	FPS
Euclidean clustering	1.74	0.21	0.77	0.14	23.3%	31
Region Growing	1.68	0.19	0.74	0.15	13.3%	37
Hierarchical Clustering	1.25	0.20	0.70	0.14	16.6%	22
Ours	0.95	0.16	0.71	0.13	6.67%	41

## Data Availability

The raw data supporting the conclusions of this article will be made available by the authors on request.
